# Data on prevalence and management practices of malaria-typhoid co-infection in Unwana South East Nigeria

**DOI:** 10.1016/j.dib.2022.108645

**Published:** 2022-09-30

**Authors:** Segun Solomon Ogundapo, Soniran Olajoju Temidayo, Karian Chigozie Ngobidi, Ibukun Caroline Vining-Ogu, Nwogo Ajuka Obasi, Victor Uzochukwu Olugbue, Lawrence Olusegun Ajala, Benneth Nnanyelugo Enemchukwu, Kalu Udo Kalu, Nnanna Oji Okoro, Ifeoma Obasi Otu, Racheal Iniobong Okon, Emelda Chinedu Ezugwu, Peace Nkeiruka Okoro, Jessica Chinagorom Onyeamachi, Ocha Udu Arua

**Affiliations:** a Department of Pharmaceutical Technology, Akanu Ibiam Federal Polytechnic Unwana Afikpo Ebonyi State, Nigeria; b Department of Science Laboratory Technology, Akanu Ibiam Federal Polytechnic Unwana Afikpo Ebonyi State, Nigeria; c Department of Medical Biochemistry, Alex Ekweme Federal University Ndufu-Alike Ebonyi State, Nigeria

**Keywords:** Malaria-Typhoid, Adherence, Prevalence, Ciprofloxacin, Management practices

## Abstract

Using a descriptive survey design, the prevalence and management practices of malaria and malaria- typhoid co-infection in Unwana South East Nigeria was determined. Two hundred and thirty-six (236) febrile volunteers comprising 104 males and 132 females attending the Medical Centre of Akanu Ibiam Federal polytechnic Unwana, Afikpo Ebonyi state Nigeria participated in this study. Using thick film microscopy and Widal antigen-based agglutination test, one hundred and thirty-seven participants were diagnosed with malaria mono infection while ninety-nine were diagnosed with malaria-typhoid co-infection. Structured questionnaire was used to obtain data on the management practices and attitudes that constitute risk factors to increased incidence of treatment failure of malaria and malaria- typhoid co-infection. The dataset [1] is relevant as a baseline and reference for further research related to factors associated with increased risk of treatment failure and emergence of drug resistance of malaria and malaria-typhoid co-infection in resource poor setting.

**Specifications Table**

**Table:** 

Subject	Epidemiology
Specific subject area	Prevalence, Prevalence, malaria-typhoid, management practices, co-infection
Type of data	Charts and Tables
How data were acquired	Data was obtained from thick film Microscopy, Widal antigen-based test kit and responses to structured questionnaire.The questionaire comprised of section one on demographic data of participants and data on knowledge attitudes and management practices of malaria and its co-infection with typhoid in the study population
Data format	Raw and analysed data expressed as tables and charts
Description of data collection	Data (malaria microscopy) and (Widal agglutination test) were obtained from analysis on blood samples collected from consenting volunteers attending the polytechnic Medical Centre presenting febrile signs and symptoms
Data source location	Akanu Ibiam Federal Polytechnic Unwana, Afikpo Ebonyi State South east Nigeria, situated on latitude 5^0^.53N and longitude 7^0^.56E.
Data accessibility	Data was deposited at Mendeley data repository DOI:10.17632/yk9dcmd94w.1 Direct URL to data: https://data.mendeley.com/datasets/yk9dcmd94w/1

## Value of the Data

• The data provided are of value as it describes the incidence, prevalence as well as the knowledge attitude and practices associated with management of malaria and its co-infection with typhoid in the study population.
• BioMedical or clinical researchers interested in epidemiology of malaria and malaria typhoid can use the data as baseline further epidemiological survey.
• This dataset provides information on the prevalence of malaria and malaria-typhoid co-infection and management practices in south east part of Nigeria which may be useful to health policy makers in the region as well as the entire the country.

## 1 Data Description

**Fig. 1:**
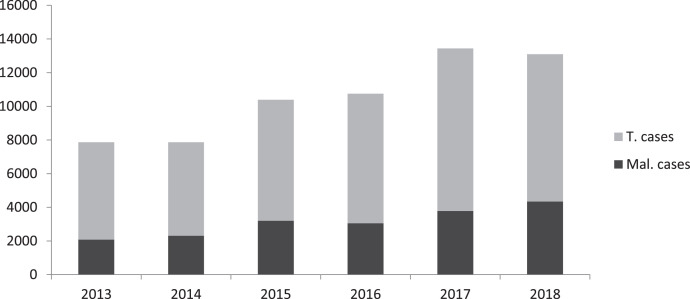
Incidence of malaria cases at the Medical Centre in the past six years (2013–2018). **(Source**: Medical Centre In/out patient's records, 2019). (**T. cases** = total number of medical cases recorded). **(M. cases** = total number of *P. falciparum* malaria cases recorded).

**Fig. 2:**
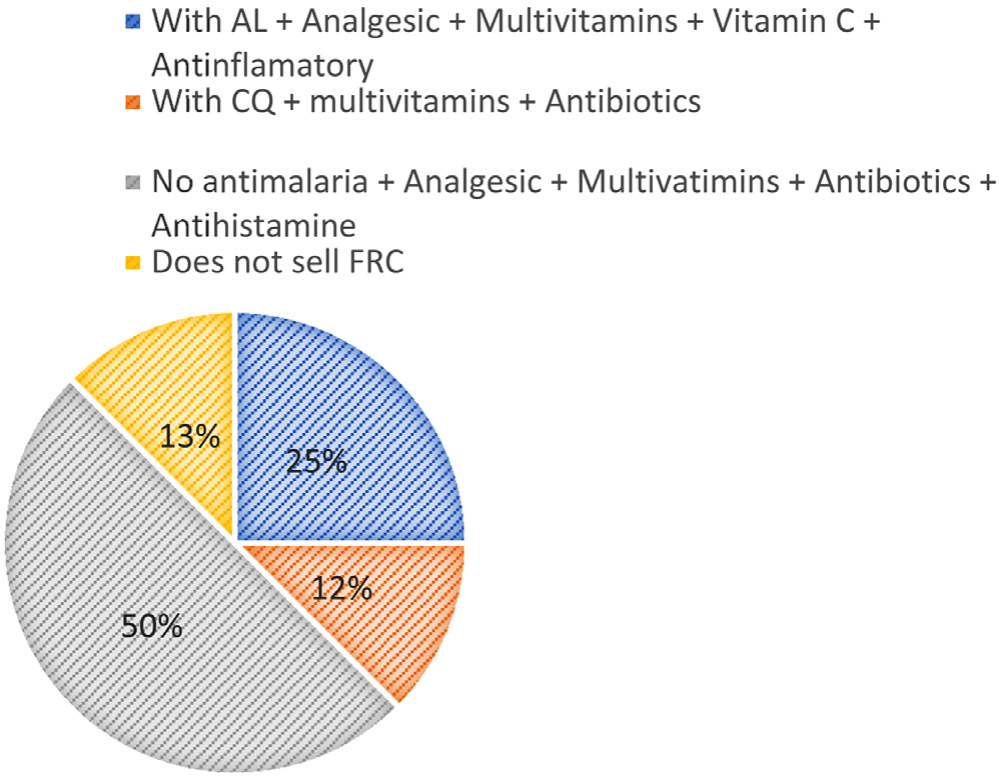
Composition of fever reducing Cocktail of drugs as sold by PPMVs.

This section consists of analysed data on parasitaemia and bacteraemia determined from blood samples obtained by vein puncture function of 236 febrile volunteers comprising 104 males and 132 females attending the Medical Centre of Akanu Ibiam Federal polytechnic Unwana, Afikpo Ebonyi state Nigeria. The analysed data of incidence of malaria in the past (2013–2018) is shown in Fig. 1, demographic characteristics of the population (Table 1), malaria parasite density Table 2, Widal titre distribution Table 3 as well as the responses to structured questionnaire on management practices of malaria and malaria-typhoid co-infection is also presented in Tables 4 and 5. Fig. 2 shows the composition of fever reducing drugs as sold by the patent Proprietary Medicine Vendors (PPMV). The raw data file has been deposited in Mendeley data repository [1]. The structured questionnaire is provided as a supplementary file in this article while the participants’ response to the questionnaire are available as Mendeley dataset [1].

**Table 1:** Baseline characteristics of study participants.

Characteristics	Malaria Infected*n* (%)	Malaria typhoid co-infected*n* (%)
SEX		
Male	67 (28.39)	37 (15.68)
Female	70 (29.66)	62 (26.27)
Total	**137 (58.05)**	**99 (41.95)**

AGE		
18-22	32 (13.60)	16 (6.78)
23-27	68 (28.81)	53 (22.46)
28-32	10 (4.24)	11 (4.66)
33-37	8 (3.39)	9 (3.81)
38-42	8 (3.39)	8 (3.39)
43-47	3 (1.27)	-
48-52	6 (2.54)	1(0.42)
52 and above	2 (0.85)	1(0.42)
Total	**137 (58.05)**	**99 (41.95)**

PROFFESSION		
Academic staff	3 (1.27)	4 (1.69)
Nonacademic staff	18 (7.63)	12 (5.08)
Students	107 (45.34)	78 (33.05)
Others	9 (3.81)	5 (2.12)
Total	**137 (58.05)**	**99 (41.95)**

*N* = 236. Values in parenthesis are % of the total number of subjects.

**Table 2:** *P. falciparum* malaria parasite density spread across the two groups.

	Participants with malaria			Participants with Co-infection		
Parasite Density/uL	Male*n*(%)	Female*n*(%)	Total*n*(%)	Male*n*(%)	Female*n*(%)	Total*n*(%)
1-999	15 (10.95)	19 (13.87)	34 (24.82)	16 (16.16)	16 (16.16)	32 (32.32)
1000-9999	45 (32.85)	42 (30.66)	87 (63.50)	19 (19.19)	32 (32.32)	51 (51.51)
>10,000	7 (5.11)	9 (6.57)	16 (11.68)	2 (2.02)	14 (14.14)	16 (16.16)
Pearson Chi-square *p*-value						
*Between the sexes	*0.684*			*0.041*		
*Between the two groups	*0.181*					

*N* = 236. Values in parenthesis are % of the total number of subjects.

**Table 3:** Widal titre distribution in the participants diagnosed with malaria-typhoid co-infection.

Widal Titre	Male*n*(%)	Female*n*(%)	Total*n*(%)
80	16 (16.16)	32 (32.32)	48 (48.48)
160	19 (19.19)	26 (26.26)	45 (45.45)
320	2 (2.02)	4 (4.04)	6 (6.06)

*N* = 236. Values in parenthesis are % of the total number of subjects.

**Table 4:** Participants’ responses on treatment, diagnosis and drug prescription.

	Malaria mono-infection			Malaria-Typhoid coinfection		
Variables	Male*n*(%)	Female*n*(%)	Total*n*(%)	Male*n*(%)	Female*n*(%)	Total*n*(%)
Previous Treatment						
Yes	63 (26.71)	69(29.26)	132(55.97)	36(15.26)	58(24.59)	94(39.86)
No	4(1.70)	1(0.424)	5(2.12)	1(0.424)	4(1.70)	5(2.12)
Frequency of Treatment						
Monthly	13(5.51)	21(8.90)	34(14.42)	6(2.54)	10(4.24)	16(6.78)
Fortnightly	5(2.12)	8(3.39)	13(5.51)	1(0.424)	7(2.97)	8(3.39)
Every two months	8(3.39)	8(3.39)	16(6.78)	5(2.12)	2(0.85)	7(2.97)
Few times in a year	28(11.87)	23(9.75)	51(21.62)	13(5.51)	28(11.87)	41(17.38)
Can't remember	11(4.66)	12(5.09)	23(9.75)	9(3.82)	9(3.82)	19(8.06)
first time	2(0.85)	–	2(0.85)	–	3(1.27)	3(1.27)
Place of purchase of drugs						
Medical Centre	13(5.51)	16(6.78)	29(12.30)	4(1.70)	12(5.09)	16(6.78)
No specific place	1(0.424)	–	1(0.424)	–	–	–
home preparation	6(2.54)	3(1.27)	9(3.82)	1(0.424)	2(0.85)	3(1.27)
pharmacy	14(5.94)	8(3.39)	22(9.33)	10(4.24)	20(8.48)	30(12.72)
PPVM	33(13.99)	33(13.99)	66(27.98)	22(9.32)	28(11.87)	50(21.2)
Diagnosis						
Blood test	11(4.66)	11(4.66)	22(9.33)	4(1.70)	14(5.94)	18(7.63)
Signs and symptoms	42(17.81)	44(18.66)	86(36.46)	28(11.87)	39(16.54)	67(28.41)
PMD	5(2.12)	3(1.27)	8(3.39)	3(1.27)	4(1.70)	7(2.97)
Signs and symptoms/Lab Test	5(2.12)	8(3.39)	13(5.51)	1(0.424)	1(0.424)	2(0.85)
Doctor (without lab Test)	4(1.70)	4(1.70)	8(3.39)	1(0.424)	4(1.70)	5(2.12)
Drug prescription						
Medical doctor	19(8.06)	25(10.60)	44(18.66)	11(4.66)	23(9.75)	34(14.42)
Patent medicine dealer	26(11.02)	24(10.18)	50(21.2)	16(6.78)	25(10.6)	41(17.38)
Self-medication	14(5.94)	9(3.82)	23(9.75)	6(2.54)	8(3.39)	14(5.94)
pharmacist	6(2.54)	6(2.54)	12(5.09)	3(1.27)	3(1.27)	6(2.54)
others	2(0.85)	6(2.54)	8(3.39)	1(0.424)	3(1.27)	4(1.70)

*N* = 236. Values in parenthesis are % of the total number of subjects.

**Table 5:** Participants’ responses on drugs, frequency of treatment and adherence to treatment regimen.

	Malaria mono-infection			Malaria-Typhoid coinfection		
Variables	Male*n*(%)	Female*n*(%)	Total*n*(%)	Male*n*(%)	Female*n*(%)	Total*n*(%)
Drug for treatment						
Artemether Lumefantrine (Al)	43(18.22)	50(21.18)	93(39.41)	–	–	–
Pyrimethamine sulphadoxine	4(1.69)	1(0.40)	5(2.12)	–	–	–
Chloroquine	3(1.27)	3(1.27)	6(2.54)	–	–	–
Herbs	6(2.54)	3(1.27)	9(3.81)	1(0.40)	2(0.85)	3(1.27)
Mixed drugs from PPMVs	10(4.23)	7(2.97)	17(7.20)	6(2.54)	13(5.51)	19(8.05)
Al and Ciprofloxacin	–	–	–	22(9.32)	34(14.41)	56(23.73)
AI and other antibiotics	–	–	–	4(1.69)	7(1.27)	11(4.66)
P. alaxin	1(0.40)	6(2.54)	7(1.27)	–	–	–
P. Sulphadoxine + Amoxicillin	–	1(0.40)	1(0.40)	2(0.85)	3(1.27)	5(2.12)
Chloroquine + Chloramphenicol	–	–		2(0.85)	3(1.27)	5(2.12)
Last Treatment						
2 Months	26(11.02)	21(8.90)	47(19.92)	17(7.20)	27(11.44)	44(18.64)
2 Weeks	21(8.90)	27(11.44)	48(20.34)	9(3.81)	16(6.78)	25(10.59)
3 Months	2(0.85)	3(1.27)	5(2.12)	1(0.40)	5(2.12)	6(2.54)
5 Months	2(0.85)	1(0.40)	3(1.27)	–	–	–
Can't remember	5(2.12)	6(2.54)	11(4.66)	4(1.69)	5(2.12)	9(3.81)
Last year	11(4.66)	12(5.08)	23(9.75)	6(2.54)	9(3.81)	15(6.36)
Adherence to Treatment						
Yes	56(23.73)	61(25.85)	117(49.58)	27(11.44)	49(20.76)	76(32.20)
No	11(4.66)	9(3.81)	19 (8.05)	10(4.23)	13(5.51)	23(9.75)
Reasons for non-adherence						
Don't need the drug again	7(1.27)	2(0.85)	9(3.81)	5(2.12)	4(1.69)	9(3.81)
Not applicable	57(24.15)	61(25.85)	118(50.00)	27(11.44)	50(21.19)	77(32.63)
Offensive smell of drugs	3(1.27)	7(1.27)	10(4.24)	5(2.12)	8(3.38)	13(5.51)

*N* = 236. Values in parenthesis are % of the total number of subjects.

## 2 Experimental Design, Materials and Methods

The protocol for data collection is based on a descriptive cross-sectional design. The main criterion for the classification of the participants into the two groups of malaria and malaria-typhoid co-infection is clinical laboratory diagnosis based on Giemsa thick blood film microscopy and Widal antigen agglutination test.

### 2.1 Area/Location

The dataset was deduced from responses to validated structured questionnaire on knowledge, attitude practices between June and November, 2019 at the Akanu Ibiam Federal Polytechnic Unwana Medical Centre located in Afikpo North Local Government Area of Ebonyi state, Nigeria. The Medical Centre serves the Medical needs of the entire polytechnic community and patients living in and around the polytechnic community. Afikpo is the second largest city in Ebonyi state and is located at latitude 5^0^.53N and longitude 7^0^.56E. Afikpo has a population of about 156,611 [2] and annual rainfall ranges from 1600 m to over 2000 m with the driest month having less than 29 m of rainfall. The average temperature in the area is highest in the month of March (84.00 F, 29.0 °C) and lowest in the month of August with 78.10 F (25.6 °C).

### 2.2 Recruitment Criteria of Participants

Participants were adults of both sexes between 18 and 60 years of age presenting symptoms like fever, head ache, vomiting, and weakness of the body, body pains and other symptoms indicating uncomplicated malaria and malaria-typhoid infection. The research goal and objectives were explained to the participants and they gave written consent before participating. Measures were taken by the researchers to ensure confidentiality to all individuals who participated. The researchers assigned codes as identification to all collected samples and the filled questionnaires.

### 2.3 Sample Collection and Administration of Questionnaire

Two milli-Litres (2 ml) of blood sample used for determination of prevalence of malaria and the co-infection with typhoid were collected from participants by vein puncture at the laboratory. This was used for through thick film microscopy as well as for Widal antigen-based agglutination tests for diagnosis of Typhoid. Structured questionnaire was validated by experts and the reliability found to be 87.34% using the test-retest method. The researchers assigned codes as identification to all the filled questionnaires.

### 2.4 Determination of Malaria Parasite Density

The determination of malaria parasite density was by Giemsa-stained thick blood films. The thick films were prepared, fixed in methanol stained with Giemsa stain and examined under the microscope using × 100 oil immersion objective lens. Determination of the level of *P. falciparum* parasitaemia was in microliter (μl) of thick blood film preparation and graded as: low + (1 to 999/μl), moderate ++ (1000 to 9999/μl) and high +++ (> 10,000 /μL) WHO [3].

### 2.5 Examination of Blood Sample using Widal Test

To determine the prevalence of typhoid, the Widal agglutination test was performed on all blood samples by the rapid slide titration method using commercial antigen suspension to detect somatic (O) and flagella (H) antigens. About 50 μl of the blood serum was placed on eight rows of circles on the test tiles and a drop of positive and negative serum suspension were placed beside each sample on the test slide. Antibody titer value ≥1: 80 against O and H antigen of *Salmonella typhi* was used as a baseline [4,5]. No stool or blood culture was carried out to validate the Widal antigen-based test.

### 2.6 Statistical Analysis

Data were processed using Microsoft Excel software and analyzed using Statistical Package for the Social Sciences (SPSS version 17.0) software. Descriptive statistics such as frequency and percentages were used to summarize participants’ demographic characteristics as well as the the responses of the participants to questionnaire variables. Data on prevalence of malaria and its co-infection with typhoid was analyzed for disparity between the sexes and malaria and malaria-typhoid co-infection groups using Chi-squared statistics. *P* < 0.05) was taken as statistically significant

## Ethics Statement

Institutional ethical clearance (AIFPU/REG/OR/102/VOL.4/416) was obtained after a review of the research protocol by the AIFPU Medical Centre board and the polytechnic Research and Ethics Committee. The institution Ethics committee directed the Medical Centre doctors, nurses and laboratory scientist to attend to participants and help collect data. Consent forms were distributed to consenting patients attending the Medical Centre out-patient department after the objectives and benefits of the study had been relayed to them. The signed or thumb-printed informed consent forms were then retrieved from the volunteer study subjects and tallied in a record book before inclusion in the study. Therefater, the participants were given the questionare to fill before seeing their physician.

## Funding

We acknowledged the funding by the Nigeria Tertiary Education Trust Fund (TETfund) Institution Based Research (IBR) grant (TETFUND/DRSS/POLY/UWANA-AFIKPO/2014/RP/VOL1). The funder had no role in design, data collection and interpretation of the findings.

## CRediT authorship contribution statement

**Segun Solomon Ogundapo:** Conceptualization, Methodology, Data curation, Writing – original draft. **Soniran Olajoju Temidayo:** Writing – review & editing, Methodology. **Karian Chigozie Ngobidi:** Methodology. **Ibukun Caroline Vining-Ogu:** Methodology. **Nwogo Ajuka Obasi:** . **Victor Uzochukwu Olugbue:** Methodology, Writing – review & editing. **Lawrence Olusegun Ajala:** Methodology, Writing – review & editing. **Benneth Nnanyelugo Enemchukwu:** Methodology, Writing – review & editing. **Kalu Udo Kalu:** Methodology. **Nnanna Oji Okoro:** Methodology. **Ifeoma Obasi Otu:** Methodology. **Racheal Iniobong Okon:** Methodology. **Emelda Chinedu Ezugwu:** Methodology. **Peace Nkeiruka Okoro:** Methodology. **Jessica Chinagorom Onyeamachi:** Methodology. **Ocha Udu Arua:** Methodology.

## Declaration of Competing Interest

The authors declare that they have no known competing financial interests or personal relationships which have, or could be perceived to have, influenced the work reported in this article.

## Data Availability

Data on Prevalence and Management Practices of Malaria-Typhoid Co-infection in Unwana South East Nigeria (Original data) (Mendeley Data).

## Appendix Supplementary materials

Image, application 1

Image, application 2
