# Supplementary material for: Data on prevalence and management practices of malaria-typhoid co-infection in Unwana South East Nigeria
Source: Data Brief. 2022 Sep 30;45:108645. doi: 10.1016/j.dib.2022.108645 (PMC9679488; doi:10.1016/j.dib.2022.108645)
Supplement: Supplementary file 2 [file mmc2.docx]

**INFORMED CONSENT FORM**

Informed consent form for the male and female patients attending the medical centre of **Akanu Ibiam Federal Polytechnic Unwana** (AIFPU) Afikpo and who we are inviting to participate in a research on malaria typhoid co-infection. The title of our research is **Effect of Staggered Dosing of Artemether Lumefantrin and Ciprofloxacin on Treatment Outcomes in Malaria Typhoid Co-Infection**.

**Principal Investigator; Segun Solomon Ogundapo, (PhD Medical biochemistry)**

Biochemistry Research Unit, Department of Science Laboratory Technology (SLT), AIFPU Afikpo Ebonyi State.

**OTHER COLLABORATORS:**

- Soniran Olajoju Temidayo (PhD, Parasitology) Biology Research Unit SLT Department AIFPU
- Olugbue V.U (PhD, Public health Microbiology) Microbiology Research Unit SLT Department AIFPU.
- Ibukun Vining-Ogu (Mrs) (MSc, Membrane Biochemistry) Biochemistry Research Unit, Department of SLT, AIFPU.
- Karian Chigozie Ngobidi (MSc, Clinical Biochemistry) Biochemistry Research Unit, Department of SLT, AIFPU.
- AIFPU Medical centre Doctors, Nurses, Pharmacist, Laboratory Scientists and Technicians

**To be carried out at the;**

- The Medical Centre and Laboratory, Akanu Ibiam Federal Polytechnic Unwana Afikpo and
- Biochemistry and Microbiology laboratories Of SLT.

**PART ONE: INFORMATION SHEET**

I am Segun Solomon Ogundapo, a teaching and research staff of the Biochemistry Research Unit, Department Of Science Laboratory Technology AIFPU. We are doing a research on malaria typhoid co-infection which is becoming prevalent in our country especially in the south East. I am going to give you information and invite you to be part of the research. You don’t have to decide in a hurry whether or not you want to participate in the study. You can talk to anyone you are comfortable with about this research before you agree to participate.

If there are words you do not understand, please be free to stop me and ask as we go through the information

**Purpose of the research**

Malaria-typhoid co-infection which is caused by infection with plasmodium spread by mosquitoes and a bacterium called *Salmonella* which we get through eating and drinking contaminated food or water, is a major public health problem affecting Africa and some other regions of the world. Among the drugs commonly taken to treat this disease are artemether lumefantrin and ciprofloxacin and they are usually taken orally together. When drugs are taken together like that, they or their products of metabolism may interact inside us. This interactions can affect the efficacy of any of the drugs, lead to drug resistance or even cause some tissue damage. The reason we are carrying out this project is to determine if spacing of the antimalaria and antibiotic is better than taking them together in terms of clearing the deadly organisms causing the disease from our body and reducing the damage to our body tissues or organs.

**Type of Research Intervention**

This research will require that we will take your blood sample (about 5ml), saliva and urine samples to carry out test to check if you have malaria or typhoid or both of them. Based on the result, the doctor will prescribe the drugs you will take and tell you how you will take them based on our study design. Seven days after your first visit to the medical centre, we would need you to come back to the medical centre to take another blood sample so that we can confirm if the dangerous agents causing the disease has been cleared from your body. Please, take note that we intend to publish the findings from this study in international peer review journal but your confidentiality will be preserved.

**Participant selection**

We are inviting all adults with fever who attend the polytechnic medical centre to participate in the research to determine if spacing of the artemether lumefantrin and ciprofloxacin when treating malaria typhoid co-infection is more effective than taking them together.

**Voluntary Participation**

Your participation in this research is entirely voluntary. It is your choice whether to participate or not. Whether you choose to participate or not, all the services you receive at this clinic will continue and nothing will change. If you choose not to participate in this research project, you will be offered the treatment that is routinely offered in this clinic/hospital for typhoid and malaria. You may change your mind later and stop participating even if you agreed earlier.

**Information on the Trial Drugs (**Artemether lumefantrin and Ciprofloxacin**)**

The drugs we are using for this research are Artemether lumefantrin (which comes with brand names like Lumartem^®^, Lokmal^®^, Coartem^®^ etc) and ciprofloxacin (which come with brand names like Ciprotab^®^ These were obtained from major distributors through the medical centre pharmacist) and they are examples of some of the commonly prescribed drugs for treatment of malaria and typhoid in our country. Please let us know if you have never taken any of them before.

The healthcare workers will be looking after you and the other participants very carefully during the study. If there is anything you are concerned about or that is bothering you about the research please talk to me or one of the other researchers.

**Procedures and Protocol**

Blood, saliva and urine will be needed from you for this study. We will take blood from your arm using a syringe and needle. Each time we will take about this much blood (about a spoonful). In total, we will take about 10mls (about 2 spoonfuls) of blood in 4 weeks. Each time we collect blood from you we will also collect early morning saliva and urine. The first samples will be collected and used to run malaria, typhoid and some biochemical tests. The second set of samples will be taken to run the same tests after completing malaria and typhoid treatment to check if you are cured. At the end of the research, after 4 weeks, any leftover blood sample will be destroyed.

**Duration**

The research will take place over a period of three months (April-June, 2019) in total. During that time, it will be necessary for you to come to the medical centre 3 times if your test result shows you have only malaria or both malaria and typhoid and you may not likely spend more than 3 hours each on each occasion you come. We would like to meet with you 4 weeks after your last clinic visit for a final check-up. \this will enable us determine if you are totally free of the infectious agents

**Side Effects**

During the course of this study, we will not give you any drug that you have not have not taken before so whatever side effects you will feel may not be different from the ones you are used to. The only difference is the time you will take the two drugs. If you feel any other type of reaction different from the ones you are used to such as making you tired or have a headache, we will follow you closely and keep track of any unwanted effects or any problems. We may use some other medicines to decrease the symptoms of the side effects or reactions. Or we may stop the use of one or more drugs. If this is necessary we will discuss it together with you and you will always be consulted before we move to the next step.

**Risks**

By participating in this research, it is possible that you will be at greater risk than you would otherwise be. There is, for example, a risk that your disease will not get better and that the new treatment regimen may not work even as well as the old one. If, however, the medicine is not working and your fever does not go down in 48 hours we will give you quinine injections which will bring your fever down and make you more comfortable.

**Benefits**

If you participate in this research, you will have the following benefits:

1. Free treatment with artemether lumefantrin and ciprofloxacin.

2. You will also be give a one malt and canned milk when you return for to check.

3. Apart from the above your participation is likely to help us find the answer to the research question. There may not be any immediate benefit to the society at this stage of the research, but future generations are likely to benefit.

**Confidentiality**

The information that we collect from this research project will be kept confidential. Information about you that will be collected during the research will be put away and no-one but the researchers will be able to see it. Any information about you will have a number on it instead of your name. Only the researchers will know what your number is and we will lock that information up with a lock and key.

**Sharing the results**

The knowledge that we get from doing this research will be shared with you through community meetings before it is made widely available to the public. Confidential information will not be shared. There will be small meetings in the community and these will be announced. After these meetings, we will publish the results in order that other interested people may learn from our research.

**Right to Refuse or Withdraw**

You do not have to take part in this research if you do not wish to do so and refusing to participate will not affect your treatment at this clinic in any way. You will still have all the benefits that you would otherwise have at this clinic. You may stop participating in the research at any time that you wish without losing any of your rights as a patient here. Your treatment at this clinic will not be affected in any way.

**Who to Contact**

If you have any questions you may ask them now or later, even after the study has started. If you wish to ask questions later, you may contact any of the following:

**Dr Segun Solomon OGUNDAPO:** Biochemistry Research Unit, Department of Science Laboratory Technology, Akanu Ibiam Federal Ppolytechnic Unwana Afikpo Ebonyi State. **08039464644.**

- Soniran Olajoju Temidayo (PhD, Parasitology) Biology Research Unit SLT Department AIFPU
- Olugbue V.U (PhD, Public health Microbiology) Microbiology Research Unit SLT Department AIFPU.
- Ibukun Vining-Ogu (Mrs) (MSc, Membrane Biochemistry) Biochemistry Research Unit, Department of SLT, AIFPU.
- Karian Chigozie Ngobidi (MSc, Biomedical and Pharmacological Biochemistry) Biochemistry Research Unit, Department of SLT, AIFPU.
- AIFPU Medical centre Doctors, Nurses, Pharmacist, laboratory scientists and technicians

**This proposal has been reviewed and approved by; Research ethics committee, School of Science Technology, AIFPU, Research Ethics committee AIFPU. These bodies’ task is to make sure that research participants are protected from harm.**

**PART II: Certificate of Consent**

**I have read the foregoing information, or it has been read to me. I have had the opportunity to ask questions about it and any questions that I have asked have been answered to my satisfaction. I consent voluntarily to participate in this research.**

**Print Name of Participant__________________**

**Signature of Participant ___________________**

**Date ___________________________**

**Day/month/year**

**If illiterate**

A literate witness must sign (if possible, this person should be selected by the participant and should have no connection to the research team). Participants who are illiterate should include their thumb-print as well.

**I have witnessed the accurate reading of the consent form to the potential participant, and the individual has had the opportunity to ask questions. I confirm that the individual has given consent freely.**

**Print name of witness_____________________ AND Thumb print of participant**

**Signature of witness ______________________**

**Date ________________________**

**Day/month/year**

I confirm that the participant was given an opportunity to ask questions about the study, and all the questions asked by the participant have been answered correctly and to the best of my ability. I confirm that the individual has not been coerced into giving consent, and the consent has been given freely and voluntarily.

A copy of this ICF has been provided to the participant.

**Print Name of Researcher****/person taking the consent________________________**

**Signature of Researcher /person taking the consent__________________________**

**Date ___________________________**

**Day/month/year**
